# Comparison of high-flow nasal cannula and conventional oxygen therapy for high-risk patients during bronchoscopy examination: protocol for a randomized controlled trial

**DOI:** 10.1186/s13063-022-07001-5

**Published:** 2023-01-05

**Authors:** Hao Qin, Guo-Qiang Jing, Wei Tan, Jun Wang, Yi-Nan Yin, Rong-Zhang Chen, Wei Zhang, Jie Li

**Affiliations:** 1 Department of Respiratory and Critical Care Medicine, Shanghai Changhai Hospital, the First Affiliated Hospital of Second Military Medical University, Shanghai, China; 2 Department of Pulmonary and Critical Care Medicine, Binzhou Medical University Hospital, Binzhou Medical University, Binzhou, Shandong China; 3 Department of Respiratory and Critical Care Medicine, the First Affiliated Hospital, China Medical University, Shenyang, China; 4 Department of Respiratory and Critical Care Medicine, West China Hospital, Sichuan University, Chengdu, China; 5 Department of Respiratory, Shanghai East Hospital, Shanghai, China; 6 Department of Cardiopulmonary Sciences, Division of Respiratory Care, Rush University Medical Center, Chicago, IL USA

**Keywords:** High-flow nasal cannula, Conventional oxygen therapy, Bronchoscopy examination, Hypoxia

## Abstract

**Introduction:**

High-flow nasal cannula (HFNC) has been proven to improve oxygenation and avoid intubation in hypoxemic patients. It has also been utilized during endoscopy examination to reduce the incidence of hypoxia. However, little is known about the effects of HFNC versus conventional oxygen therapy (COT) on oxygenation during bronchoscopy examination via nasal route; particularly, no study has compared the use of HFNC with that of COT at similar F_I_O_2_ for patients who have high-risk factors of desaturation during bronchoscopy examination.

**Methods and analysis:**

This randomized controlled trial will be implemented in four academic centers in China. Patients who have high-risk factors including hypoxemia, hypercapnia, morbid obesity, and narrow airway will be enrolled to use HFNC or COT during bronchoscopy examination. In the HFNC group, the initial gas flow will be set at 50 L/min with a fraction of inspired oxygen (F_I_O_2_) at 0.45, if the patient tolerates, the flow can be increased to 60L/min at most, while in the COT group, oxygen flow will be set at 6 L/min via a conventional nasal cannula. After 5 min pre-oxygenation, the bronchoscope will be inserted via the nasal route. Vital signs, oxygenation (SpO_2_), and transcutaneous CO_2_ (PtCO_2_) will be continuously monitored. The primary outcome is the incidence of hypoxemia, defined as SpO_2_ < 90% for 10 s during bronchoscopy examination, and secondary outcomes include the need for treatment escalation and adverse events.

**Discussion:**

Hypoxia is a common complication of bronchoscopy, our study attempted to demonstrate that HFNC may reduce the probability of hypoxia during bronchoscopy in high-risk patients. The results will be disseminated through peer-reviewed journals and national and international conferences.

**Trial registration:**

http://www.chictr.org.cn/: ChiCTR2100055038. Registered on 31 December 2021.

## Strengths and limitations of this study

### Strengths

- ➣ This is the first study to compare the utilization of HFNC with that of conventional nasal cannula at similar F_I_O_2_ during bronchoscopy examination via nasal route for patients who have high-risk factors
- ➣ This is the first study to report the need for treatment escalation to resolve hypoxemia during bronchoscopy examination, of which the study findings have clinically meaningful implications
- ➣ This is the first study to demonstrate the effects of HFNC on transcutaneous CO_2_ during bronchoscopy examination.

### Limitations

- ➣ The study enrolls patients with four different risk factors; the general conclusion might not apply to any specific group.
- ➣ This study does not limit the procedures of bronchoscopy; the general conclusion might not apply to any specific procedure.
- ➣ This study excludes critically ill patients, thus the findings may not apply to these patients

## Introduction

### Background

High-flow nasal cannula (HFNC) is an oxygen supply system that allows setting the gas flow and the fraction of inspired oxygen (F_I_O_2_) separately [1]. Because of its ability to provide high gas flow to exceed the inspiratory flow demand of patients, F_I_O_2_ is constant during HFNC treatment [2, 3]; moreover, the high flow also generates positive end-expiratory pressure [1, 4]. Therefore, HFNC has been proven to be superior to conventional oxygen therapy (COT) in improving oxygenation and avoiding intubation in hypoxemic patients [5–14]. Additionally, HFNC is also shown to reduce hypercapnia and improve ventilation in patients with chronic obstructive pulmonary disease (COPD) [15–21], since high-flow gas washes out the dead space [19–21].

In recent years, HFNC has been utilized to improve oxygenation or avoid desaturation during endoscopy examination [22–37], as hypoxemia is the most common complication during endoscopy examination, particularly with sedation [31–33, 38, 39]. The reported incidence of hypoxemia in the RCTs varied from 28.8 to 89.7% during flexible bronchoscopy examination [22–30]. Patients who require bronchoscopy examination may have existing pulmonary diseases, and the insertion of bronchoscope occludes part of the airway, resulting in increased respiratory resistance. Both factors may contribute to hypoxemia and hypercapnia during bronchoscopy examination [40–42].

Till now, seven randomized controlled trials (RCTs) have compared the use of HFNC with that of COT during bronchoscopy examination and found that the incidence of hypoxia was lower in the HFNC group [22–26, 29, 30]. Most of the RCTs [22, 25, 26, 29, 30] were implemented with bronchoscopy via the oral route, and during the examination, HFNC was utilized with the mouth constantly open, which might cause the significant loss of the benefits of HFNC in constant F_I_O_2_ and positive end-expiratory pressure. Nasal insertion of bronchoscope would allow patients to breathe with mouth closed, which might reserve the benefits of HFNC. Besides, nasal insertion of bronchoscope is more favorable in our institutions, because it causes fewer retch reflexes and enables better control during insertion as well as additional inspection of the nasal airway [42]. Nevertheless, little evidence is available to support the utilization of HFNC during bronchoscopy via nasal insertion. Therefore, we propose an RCT to compare the use of HFNC versus that of COT during bronchoscopy examination via nasal insertion among high-risk patients. Besides hypoxemic patients, we will also enroll three other types of patients as “high-risk” patients, including (1) patients of hypercapnia with chronic pulmonary disease, who might have increased CO_2_ and hypoxemia during bronchoscopy [40–42], (2) morbidly obese patients who were reported to experience hypoxemia during endoscopy examination [32], and (3) patients of narrow airway with radiology evidence prior to bronchoscopy [42, 43]. Our hypothesis is that the incidence of hypoxemia will be lower in the HFNC group than in the COT group during nasal bronchoscopy examination for patients with high-risk factors.

## Methods

This is a multi-center randomized controlled trial, which has been approved by the Ethic Committees of East Hospital (Shanghai, China) and Changhai Hospital (Shanghai, China). This trial is registered with Chictr.org.cn (ChiCTR2100055038). The report of the protocol followed the SPIRIT guideline [44].

### Study population

Patients who need bronchoscopy examination will be recruited from outpatient clinics or inpatient units in the four academic hospitals in China.

#### Inclusion criteria

Adult patients who have one of the following high-risk factors will be enrolled in the study: (1) hypoxemia with the PaO_2_/F_I_O_2_ ≤ 300 mmHg before bronchoscopy examination; (2) hypercapnia with PaCO_2_ ≥ 45 mmHg and baseline chronic pulmonary disease, including COPD, and bronchiectasis, etc.; (3) narrow trachea confirmed by radiology before bronchoscopy, which may have been caused by tracheomalacia, trachea tumor or granuloma, or aspirated foreign body, etc.; and (4) morbidly obese patients with body mass index (BMI) ≥ 30 kg/m^2^.

#### Exclusion criteria

Patients will be excluded if any of the following criteria is met: (1) refuse to participate; (2) local/general anesthesia bronchoscopy or rigid endoscopy is indicated; (3) age ≥ 90 years, or < 18 years; (4) pregnancy; (5) estimated duration of bronchoscopy is less than 10 min; (6) contraindication to using HFNC, including nasopharyngeal obstruction and blockage; (7) require oxygen flow ≥ 3L/min to maintain SpO_2_ at 90–97%; (8) central airway narrowness > 80%; and (9) the patient is critically ill and unsuitable for study inclusion assessed by anesthesiologists and pulmonologists.

### Study procedures

**Fig. 1:**
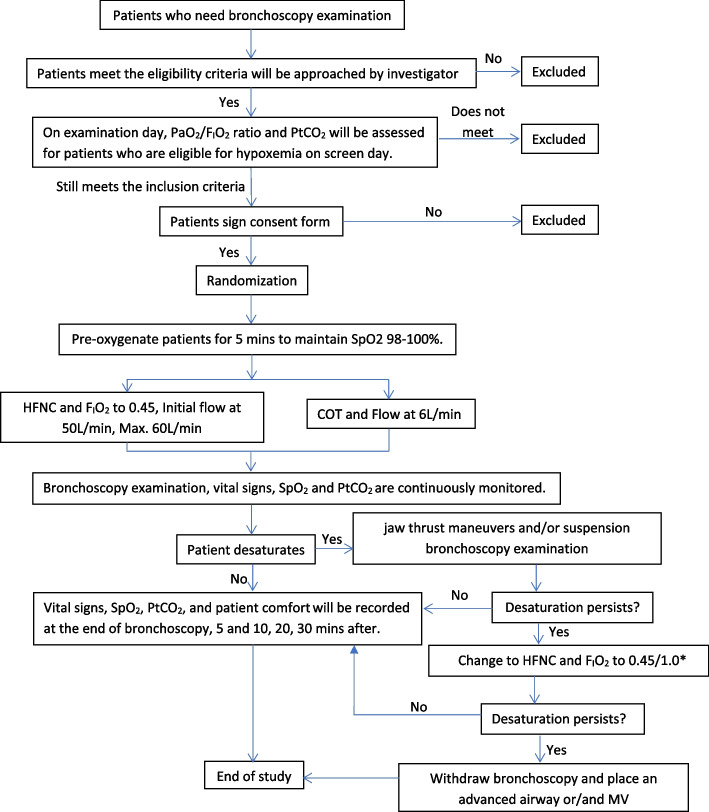
Study flowchart. Abbreviations: HFNC, high-flow nasal cannula; HR, heart rate; RR, respiratory rate; BP, blood pressure; SpO_2_, peripheral capillary oxygen saturation; PtCO_2_, percutaneous carbon dioxide; MV, mechanical ventilation. ^*^For HFNC group, increase F_I_O_2_ to 1.0; For COT group, change to HFNC at 60 L/min and F_I_O_2_ 0.45, if SpO_2_ cannot be maintained, increase F_I_O_2_ to 1.0

See the study diagram in Fig. 1.

#### Recruiting and consent process

The nurse responsible for scheduling bronchoscopy examinations for patients in the bronchoscopy suite will screen the patients. The study investigator will be notified of eligible patients and will obtain informed consent from the patients. Study purposes, procedures, risks, and benefits will be discussed with the patients in a private room. Consent forms will be given to patients for full consideration before the examination day. On the examination day, arterial blood gas will be assessed for patients who are enrolled in the study due to hypoxemia and/or hypercapnia on the screen day. If the patients still meet the inclusion criteria and sign the consent form, they will be enrolled for randomization. Together with the other two types of patients (narrow airway and morbidly obese), they will be randomized to receive HFNC or COT during bronchoscopy examination after signing the consent form in the presence of the study investigator.

#### Randomization and treatment

An independent statistician will be responsible for generating the randomization sequence, which will be placed in a series of sequentially numbered, sealed, and opaque envelopes. After the patient signs the consent form, an envelope will be opened to assign an oxygen device for the patient.

OH-70C (micomme, HuNan, China) will be used to provide HFNC, with initial flow of 50 L/min and F_I_O_2_ at 0.45. The temperature will be set at 37° C. Nasal cannula will be chosen based on the patient’s prong size: the nasal cannula size should be less than 50% of the nasal prong size. Similarly, in the group with COT, a double-prong nasal cannula (Healthcare Medical Supplies Co., Ltd. Hangzhou, China) will be used, and oxygen flow will be set at the maximum setting of 6 L/min, in which F_I_O_2_ is calculated to be 0.45. Before using the assigned device, pre-oxygenation will last for 5 min to maintain SpO_2_ 98–100% by using an anesthetic mask, due to the need for adjusting the sedation dose. Duration of pre-oxygenation, patient SpO_2_, PtCO_2_, and vital signs including heart rate, respiratory rates, and blood pressure before and after pre-oxygenation will be recorded (Fig. 1).

#### Bronchoscopy examination and hypoxemia management

Topical anesthesia will be provided prior to bronchoscopy as routine treatment. Sedation and/or anesthesia will be utilized by an anesthesiologist at the bedside. The depth of sedation will be assessed using the Modified Observer’s Assessment of Alertness/Sedation Scale(MOAA/S), and the score will be maintained at 2 ~ 3 throughout the examination process. The timing to use, the name, and the dose of sedation will be recorded. Bronchoscopy examination will be performed by experienced pulmonologists in the bronchoscopy suite. SpO_2_, PtCO_2_, and vital signs will be continuously monitored while F_I_O_2_ for HFNC and oxygen flow for COT will be maintained until the patients experience the first desaturation, defined as SpO_2_ ≤ 90% for 10 s. The timing and the lowest SpO_2_ in the first desaturation will be recorded (Fig. 1). Once desaturation occurs, jaw thrust maneuvers and/or suspension of bronchoscopy examination will be implemented to improve oxygenation. If desaturation persists, F_I_O_2_ will be increased to 1.0 in the HFNC group while in the COT group, COT will be changed to HFNC with flow at 60 L/min and initial F_I_O_2_ at 0.45, F_I_O_2_ will be increased to 1.0 if hypoxia remains. All the management will be recorded as treatment escalation. If SpO_2_ is still below the safe range, advanced airways, including nasopharyngeal airway, oropharyngeal airway, laryngeal mask, or tracheal intubation, will be inserted depending on patient situation (Fig. 1). The process, the duration, and the reason for the suspension of bronchoscopy examination will be recorded. After the recovery, whether bronchoscopy will be resumed or not will be determined by pulmonologists based on the clinical situation. Notably, besides desaturation, other adverse events may also cause the examination to be suspended, such as tachycardia, severe hypertension, arrhythmia, etc. These events will also be recorded in detail. The duration of the bronchoscopy examination refers to the time from the first entry into the nasal cavity to the end of the examination, as well as the time of per interruption will be precisely documented.

#### Termination criteria

Bronchoscopy examination will be terminated if any of the criteria is met: (1) any of the adverse events, including hemodynamic instability, cardiac arrest, massive bleeding, and pneumothorax is observed or reported, and (2) despite maximal oxygen delivery, SpO_2_ cannot be maintained at a level ≥ 90%.

All the rescue treatments and resuscitation equipment are prepared in the bronchoscopy suite. Any patient who experiences adverse events will be rested and monitored in the general unit, until the patient recovers.

#### Post bronchoscopy monitoring

After bronchoscopy, SpO_2_, PtCO_2_, and vital signs will be continuously monitored until the patients return to pre-bronchoscopy status or are stable. The recovery time and postoperative adverse reactions will be recorded as well. After the patients recover, patient comfort will be assessed. Lastly, we will also survey the anesthesiologists and pulmonologists on their satisfaction with the bronchoscopy examination.

### Outcomes

The primary outcome is to compare the incidence of desaturation which is defined as SpO_2_ < 90% between patients using HFNC and those using COT. The secondary outcomes include the timing of the appearance of the first desaturation (namely desaturation time), the lowest SpO_2_ and the highest PtCO_2_ during the entire bronchoscopy examination, the need for treatment escalation and airway management, the number and duration of bronchoscopy suspensions, the incidence of adverse events, and the recovery time between the two groups. A post hoc analysis will be performed to explore the risk factors of desaturations during bronchoscopy examination among high-risk patients.

### Sample size calculation (https://clincalc.com/stats/samplesize.aspx)

This study is a superiority study. While in the study [26] with a patient population close to our study during bronchoscopy examination, the incidence of desaturation of HFNC vs COT group was 13.3 vs 33.3%, respectively. However, this previous study only included hypoxemic patients while we will enroll other types of patients, so the incidence of hypoxemia in the HFNC group in their study might be higher than the incidence in ours. Thus, we assumed that when the maximal concentration of oxygen is provided by the two assigned devices, that is, when F_I_O_2_ is set at 1.0 for HFNC and oxygen flow at 6 L/min for COT, the probability of desaturation is 10% in the HFNC group and 30% in the COT group, respectively. According to statistical calculation, the sample size should be 124, with a confidence level (α) of 95%, power (1-ß) of 80%, and margin (Δ) of 0.2. Assuming a 20% dropout rate during the study, we set the final sample size to be 148. An interim analysis will be conducted once 50% of the sample size is reached.

### Data collection

The parameters that are continuously monitored by the bedside monitor before, during, and after bronchoscopy examination, including vital signs, SpO_2_, and PtCO_2_, and the incidence of desaturation and bronchoscopy suspension/termination will be observed and recorded by an independent investigator. Meanwhile, the anesthesia information and procedures under bronchoscopy such as bronchoalveolar lavage, EBUS, and biopsy, as well as bleeding volume and the adverse events during the examination, will be collected. Demographic information of patients (age, gender, height, weight, BMI, race, smoking and drinking history, diagnosis, comorbidity, current medication, and reason for bronchoscopy examination) and laboratory results such as coagulation and chest CT will also be collected.

### Statistical analysis

Categorical variables, including the incidence of desaturation, treatment escalation, suspension or termination of bronchoscopy, etc., will be presented as percentages and analyzed by chi-square test. Continuous variables, including the desaturation time, recovery time, the lowest SpO_2_, the highest PtCO_2_, etc., will be presented as mean ± standard derivation (SD) or median and interquartile rage (IQR) and compared by independent *t*-test or Mann Whitney test, depending on the normality of distribution, which will be tested by the Kolmogorov–Smirnov test. A two-sided *P*-value of < 0.05 will be regarded as statistically significant for all tests. Data analysis will be conducted with SPSS software (SPSS 23.0; Chicago, IL).

### Patient and public involvement

Patients and/or the public were not involved in the study design.

## Discussion

This is the first study to compare HFNC vs COT during bronchoscopy via nasal route among high-risk patients. The benefit of using the nasal route is that it allows for better control of the bronchoscope [42]. More importantly, because the patient’s mouth is mainly closed during the examination, the placement of HFNC may help generate and maintain the positive end-expiratory pressure, resulting in better oxygenation during HFNC. Even though NIV has been shown to be more effective in improving oxygenation during bronchoscopy examination, the complications of NIV, particularly gastric overdistension, are concerning [45], since in this process the bronchoscope is inserted via the oral route. In patients who receive positive pressure ventilation with the mouth constantly open and a bronchoscope is inserted via the bite block, the closure of vocal cords and the function of swallow will be substantially affected, and the risk of aspirating gas from positive pressure ventilation might be higher than in HFNC. Therefore, although we will not compare HFNC with NIV in this study, when HFNC is placed in two prongs at the same flow with the mouse closed, the nasal route may generate positive pressure comparable to that in NIV and produce similar washing-out effects [46, 47].

We only enroll patients with high-risk factors, due to the consideration of cost-effectiveness and clinical implication. For the general patient population, the incidence of desaturation during endoscopy examinations is low. Even when hypoxemia occurs during bronchoscopy examination, most can be easily resolved by increasing oxygen flows through the conventional nasal cannula. More importantly, since a general bronchoscopy examination usually lasts 10–15 min, using a new set of HFNC circuits and nasal cannula might not be worthwhile, especially considering the high cost of HFNC, which is 200–400-fold of conventional nasal cannula. As such, using HFNC to assist bronchoscopy examination for patients with high-risk factors might be more reasonable [48].

Moreover, Riccio et al. compared nasal cannula with HFNC at similar F_I_O_2_ during colonoscopy examination in morbidly obese patients and did not find a significant difference between the two groups, which implies the F_I_O_2_ other than high-flow setting is essential to the maintenance of oxygenation during endoscopy [32]. However, Yilmazel Ucar E et al. [25] compared the use of HFNC with that of COT at the same F_I_O_2_ during the endobronchial ultrasonography (EBUS) procedure among 170 patients and found a lower incidence of desaturation with HFNC. They did not enroll any patients with high-risk factors, so the exact effect of flow settings on oxygenation during bronchoscopy for patients with high-risk factors is still unclear. More importantly, in our study, we plan to compare HFNC and COT at similar F_I_O_2_ (~ 0.45), which is the maximum F_I_O_2_ setting for the COT group. If patients in the COT group continue to desaturate, they will be switched to HFNC with F_I_O_2_ at 1.0. Advanced airways will be inserted if the oxygenation still cannot be maintained. This management of hypoxemia and the study findings have an important clinical implication, as the consequences of bronchoscopy suspension or the need to switch to a different oxygen device is more meaningful than the incidence of desaturation, which might be easily resolved by simply increasing the oxygen flow with a standard nasal cannula, especially in the studies using low flow (2–3 L/min) oxygen in the COT group [23, 24, 29].

One may concern about hypercapnia during bronchoscopy [46], due to the partial occlusion of the airway and the side effects of sedation, so our study will continuously monitor PtCO_2_ before, during, and after bronchoscopy, which is also the first clinical study reporting the effects of HFNC on PtCO_2_ during bronchoscopy examination.

The major limitation of this study is the difficulty of excluding the impact of the length of examination and procedures on hypoxemia. Special operations under bronchoscope (such as EBUS), long operation time, or more than 3 biopsies under bronchoscope may cause hypoxia. Even though the patients will be randomized, those factors might be balanced into two groups, and the post hoc analysis will be performed, the conclusion from this study still might not apply to any specific type of procedure during bronchoscopy examination. Additionally, it is an open-label study, as the intervention (HFNC) is a unique device, which is largely different from the conventional nasal cannula, thus blinding the participants or investigators is impossible.

## Trial status

We originally planned to start enrolling patients on June 1, 2022, and end the study within 1 year, but due to the COVID-19 outbreak in China, it may be delayed.

## Data Availability

Data will be sourced from forced expiratory capacity test results, electronic medical records, and monitors. Data will be de-identified and entered into a secure, web-based electronic database. Only the investigators will have access to the final dataset. Individual participant data after de-identification that underlie the reports reported in this article can be obtained by contacting the corresponding author. Data will be available immediately following publication and ending in 5 years.

## Abbreviations

HFNC: High-flow nasal cannula
COT: Conventional oxygen therapy
F_I_O_2_: Fraction of inspired oxygen
PtCO_2_: Transcutaneous CO_2_
COPD: Chronic obstructive pulmonary disease
ERCP: Endoscopic retrograde cholangiopancreatography
BMI: Body mass index
EBUS: Endobronchial ultrasonography
